# Depression and Its Association with Health-Related Quality of Life in Postmenopausal Women in Korea

**DOI:** 10.3390/ijerph15112327

**Published:** 2018-10-23

**Authors:** Hyejin Park, Kisok Kim

**Affiliations:** 1 Department of International Healthcare Administration, Daegu Catholic University, Gyeongsan 38430, Korea; hjpark@cu.ac.kr; 2 College of Pharmacy, Keimyung University, Daegu Metropolitan 42601, Korea

**Keywords:** demographic factor, EQ-5D, health-related quality of life, depression

## Abstract

Menopause is associated with depressive symptoms that can significantly affect a woman’s quality of life. The objective of this study was to evaluate the association between depression and health-related quality of life (HRQoL) in postmenopausal women. In this cross-sectional descriptive study, participants (*n* = 3860) were selected from the 2013–2015 Korea National Health and Nutrition Examination Survey (KNHANES). The sociodemographic characteristics, medical history of depression, and EQ-5D scores of the participants were obtained from the KNHANES dataset. Age, educational level, and income were associated with HRQoL in these participants. Moreover, depression exerted a considerable influence on HRQoL in postmenopausal women. The adjusted odds ratios in participants with depression for the EQ-5D dimensions were as follows: 5.52 (95% CI = 4.04–7.55, *p* < 0.001) for anxiety/depression, 3.86 (95% CI = 2.78–5.36, *p* < 0.001) for usual activities, and 2.52 (95% CI = 1.68–3.78, *p* < 0.001) for self-care. Our findings suggest a strong association between depression and HRQoL. Hence, preventing the onset or exacerbation of depression may significantly improve quality of life in postmenopausal women.

## 1. Introduction

The prevalence of depression is increasing worldwide [1]. Indeed, depression is one of the most common mental disorders among elderly people (prevalence, 1–16%) [2]. Along with somatic illness, functional disability, cognitive impairment, and lack of social contacts, female gender is also associated with depressive disorders in elderly populations [3]. Epidemiological and clinical studies have shown that the prevalence of depression is higher in women than in men [4] by up to twofold [5]. This difference is thought to be related to changes in endocrines that control the reproductive system [6]. Postmenopausal women experience considerable biological and psychological changes, including a decreased level of estrogen, which may be related to depression [7,8]. Estrogen interacts with its receptors in the limbic area of the brain, which is important for the regulation of emotions, cognition, and behavior [9,10].

Depression reduces an individual’s mental and physical health [11,12] and is associated with a diminished quality of life (QoL) [13,14]. Depression is also associated with several functional disturbances and significant reductions in several aspects of QoL, including social functioning [15]. In addition, depressive symptoms have various effects on physical and mental health-related quality of life (HRQoL) of elderly individuals [16]. In older adults, lowered QoL has been reported to be greatly dependent on reduced physical function. Inability to perform activities of daily living or instrumental activities of daily living has been known to be associated with decreased QoL [17,18]. Besides, depression has been found to be significantly correlated with functional disability [19]. It is known that the effect of depression on functional disability may partially be due to deteriorated physical activity and social interactions of depressed elderly individuals [20]. Depressive symptoms and disorders are frequent causes of emotional and physical suffering and are associated with elevated risks of disability in diverse areas of functioning [21].

HRQoL is influenced by sociodemographic factors, such as gender, age, educational level, and income [22]. In addition, low socioeconomic status increases the risk of depression [23]. Therefore, it is important to analyze the relationship between depression and HRQoL in postmenopausal women according to sociodemographic factors.

Few studies have addressed depression in relation to HRQoL in postmenopausal Korean women using a population-based sample. Therefore, in this report, we investigated the association between depression and HRQoL in postmenopausal women using data from the Korea National Health and Nutrition Examination Survey (KNHANES). We also evaluated the prevalence of depression according to participants’ sociodemographic characteristics and the relationship between depression-related morbidity and the five dimensions of EuroQoL. We postulate that the prevalence of depression and HRQoL may be significantly influenced by sociodemographic factors. Additionally, the prevalence of depression could be associated with HRQoL in postmenopausal women in Korea.

## 2. Methods

### 2.1. Study Population

This study used data from the KNHANES 2013–2015, which included a health and nutrition survey and a medical examination. The KNHANES sample was chosen using a stratified multistage cluster sampling method with proportional allocation based on the National Census Registry. Face-to-face interviews using a structured questionnaire were conducted by trained interviewers. From this sample, postmenopausal women were selected for inclusion in the present study. Of the postmenopausal women, 3860 provided data with no missing variables. The study protocol was approved by the Korean Ministry of Health and Welfare (# 2013-07CON-03-4C, 2013-12EXP-03-5C) and was conducted in accordance with the Ethical Principles for Medical Research Involving Human Subjects, as defined by the Helsinki Declaration. All participants in this study provided written informed consent.

### 2.2. Source of Data and Variable Definitions

Participants from selected census blocks provided information on their age, educational level, income, and medical history of depression. Height and weight were measured with the participants dressed in light clothing with no shoes to determine body mass index (BMI). BMI was calculated as weight (kg) divided by the square of height (m^2^). The participants were classified as underweight (BMI < 18.5), normal (18.5 ≤ BMI < 22.9), overweight (23.0 ≤ BMI < 24.9), or obese (BMI ≥ 25.0) according to the WHO definitions for Asian populations. In this study, HRQoL was treated as the dependent variable that was influenced by depression as an independent variable. The depression criterion was a self-reported history of physician-diagnosed depression (DSM-IV). In this study, we present the prevalence of current physician-diagnosed depression. As confounding factors, educational level and income were used as indicators of socioeconomic status. Educational level was classified as less than a middle school graduate, middle school graduate, or high school or higher. Income was calculated by dividing household income by the square root of the number of members in the household according to the Organization for Economic Co-operation and Development (OECD) method. Income was categorized into quartiles based on the income of the participant’s age group. HRQoL was assessed using the EQ-5D questionnaire. The EQ-5D is a self-reported, descriptive health status instrument with five health dimensions: mobility, self-care, pain/discomfort, usual activities, and anxiety/depression. Each dimension has three levels, namely “no problems”, “some problems”, and “severe or extreme problems” [24]. The five dimensions of the ED-5D are then converted into EQ-5D index scores using Korean specific preference weight [25]. Average scores of the EQ-5D index ranged from −0.17 to 1, where 1 indicates no problem in any of the five dimensions, zero indicates death, and negative values indicate health statuses worse than death. The Korean versions of the EQ-5D tools have been validated in a previous study [26]. The kappa value of EQ-5D in dimensions between test and retest was 0.32–0.64, and the intraclass correlation coefficient of EQ-5D was 0.61 [26]. For the purpose of this study, the levels were used as an overall measure of the perceived HRQoL.

### 2.3. Statistical Analyses

Differences in categorical variables between groups were evaluated by the Mantel–Haenszel chi-squared test. The presence of a linear trend was evaluated by defining a linear contrast in the linear regression models. Logistic regression models were used to estimate the odds ratio (OR) and 95% CIs for abnormal (disability) versus normal (no problem) in the EuroQol categories among participants who reported having depression compared with those who did not. Statistical analyses were conducted using SAS version 9.4 (SAS Institute, Cary, NC, USA).

## 3. Results

Table 1 shows the prevalence of depression according to the demographic characteristics of the participants. The prevalence of depression was in the range of 2.7–7.2%. Educational level and income level were significantly associated with depression. The prevalence of depression decreased significantly with increasing income and educational level (*p* < 0.01).

The mean EQ-5D scores of the participants are listed in Table 2. The EQ-5D score decreased with increasing age (*p* for trend < 0.001). In addition, the EQ-5D scores increased with increasing BMI (0.90, 0.89, 0.86, and 0.83 for obese, overweight, normal weight, and underweight, respectively). The EQ-5D scores also increased with increasing educational level (*p* for trend < 0.001) and income level (*p* < 0.001).

Table 3 shows the participants’ age, BMI, and EQ-5D score according to depression category. The mean age, BMI, and EQ-5D score of participants with depression were 64.7, 24.2, and 0.88, respectively. The mean age and BMI were not significantly different between participants with and without depression, whereas the EQ-5D score was significantly lower in the participants with depression (0.77 and 0.89, respectively; *p* < 0.001).

Table 4 shows the ORs for disability. The adjusted ORs for disability for each EuroQol category were significantly related to the prevalence of depression after adjusting for age (model 1) or for all other potential covariates (model 2) (*p* < 0.001). Compared to participants without depression, the adjusted ORs for mobility, self-care, usual activity, pain/discomfort, and anxiety/depression were 2.49 (95% CI, 1.79–3.47), 2.52 (95% CI, 1.68–3.78), 3.86 (95% CI, 2.78–5.36), 2.14 (95% CI, 1.57–2.92), and 5.52 (95% CI, 4.04–7.55), respectively, in those with depression (*p* < 0.001) (model 2).

## 4. Discussion

In this large population-based study, we investigated the association between depression and HRQoL using the EQ-5D. Our results indicate a significant association between depression and educational level and income level in postmenopausal Korean women. The relationship between depression and sociodemographic characteristics, such as gender, age, marital status, income level, and educational level has been investigated worldwide in various populations [27]. In many of these studies, the educational level and income of the participants were significantly related to the prevalence of depressive symptoms. The association between educational level and depression may be caused by reduced access to information about risk factors [28,29]. Alternatively, a poor education could be associated with low income, which could affect physical and mental health [30].

Our results showed that age, educational level, and income are associated with HRQoL. This is consistent with two previous studies of adult populations [31,32]. The sociodemographic determinants of HRQoL in the general population have been documented in Sweden, the Netherlands, Norway, the United Kingdom, the United States, and Japan [33,34,35,36,37,38]. In these studies, sex, age, educational attainment, income, and chronic disease had significant impacts on participants’ QoL.

In this study, depression was significantly related to HRQoL in postmenopausal women. Previous studies have found robust association between depression and low HRQoL in specific population subgroups [39,40,41,42]. In particular, several studies worldwide have explored the relationship between depressive symptoms or a depressive disorder and HRQoL in older adults. A recent study on Portuguese elderly population showed that older adults with depression symptoms had a higher probability of reporting lower levels of HRQoL after adjustment for sex, age, region, and number of noncommunicable diseases [43]. In a similar context, a review paper by Sivertsen et al. indicated a clear and consistent association between depression and lower HRQoL in older adults in clinical and community settings [44]. Based on 19 cross-sectional studies, this review found a significant association between severity of depression and lower HRQoL in older adults, regardless of assessment instruments used for HRQoL. Furthermore, in 10 longitudinal studies, a depressive disorder and a higher depressive symptom score were consistently associated with lower HRQoL, and this association was found to be stable over time. Participants with a depressive disorder at baseline had lower HRQoL at follow-up than participants without depression, and the severity of depressive symptoms at baseline had a significant effect on any improvement in HRQoL at follow-up.

Thus far, there have only been a few studies that have investigated this relationship in a population-based sample of postmenopausal women. Depression at any time of life, including postmenopausal period, is known to negatively impact QoL measures as well as somatic complaints [45]. Community-based longitudinal studies have reported that the risk of depression is significant increased during the menopause transition compared with premenopausal and that the prevalence of depression in some premenopausal women could be an important source of variability in measures of QoL at this stage of a woman’s life [45,46,47]. In our study, the ORs for disability in the five dimensions were significantly increased among postmenopausal women with depression. Anxiety/depression and usual activities exerted the greatest influence on HRQoL among postmenopausal women with depression. These results suggest that in addition to anxiety/depression, limitations on one’s usual activities are also important targets for improving HRQoL in postmenopausal women.

This study has several limitations. First, the results only indicate associations and cannot be used to establish causal relationships due to the cross-sectional design of this study. Second, self-reports of current depression state and sociodemographic variables may lead to misclassification and recall bias. Third, the use of EQ-5D to assess QoL may also have limitations related to the reliability and objectivity of the findings. However, this study also has several strengths. To our knowledge, this is the first population-based study to assess the association between depression and HRQoL among postmenopausal Korean women using nationally representative data. This study analyzed a large population based on systematic sampling, which enhances the generalizability of the findings. Investigation of factors related to HRQoL in specific subgroups is an important concern for health policymakers and for the development of appropriate interventions to improve individuals’ QoL. Our results suggest that health policymakers should focus on the effects of sociodemographic factors and depression on the HRQoL of postmenopausal women in Korea.

## 5. Conclusions

In this population-based study, HRQoL among postmenopausal women was associated with educational level and income level. In addition, postmenopausal women with depression had a significantly lower HRQoL. Depression significantly increased the ORs for disability in all five dimensions of the EQ-5D. Our results indicate a significant association between depression and HRQoL in postmenopausal Korean women. Therefore, prevention and management of depression are important for improving the QoL of postmenopausal women.

## Figures and Tables

**Table 1:** Prevalence of depression in postmenopausal women according to demographic characteristics.

Variable	*n*	Depression (%)	*p*-Value ^a^
Age (years)			
<60	1303	46 (3.5)	0.092
60–69	1255	68 (5.4)	
≥70	1302	64 (4.9)	
BMI (kg/m^2^)			
<18.5	92	4 (4.4)	0.133
18.5–22.9	1391	57 (4.1)	
23.0–24.9	990	43 (4.3)	
≥25.0	1387	74 (5.3)	
Education			
≤Elementary school	2126	112 (5.3)	0.007
Middle school	601	31 (5.2)	
≥High school	1133	35 (3.1)	
Income			
Quartile 1 (lowest)	919	66 (7.2)	<0.001
Quartile 2	980	45 (4.6)	
Quartile 3	976	40 (4.1)	
Quartile 4 (highest)	985	27 (2.7)	

Note: ^a^ *p* is determined by Mantel–Haenszel chi-squared test.

**Table 2:** Mean EQ-5D scores of postmenopausal women according to demographic characteristics.

Variable	*n*	EQ-5D	*p* for Trend ^a^
Age (years)			
<60	1303	0.94 ± 0.11	<0.001
60–69	1255	0.89 ± 0.15	
≥70	1302	0.81 ± 0.20	
BMI (kg/m^2^)			
<18.5	92	0.83 ± 0.22	0.119
18.5–22.9	1391	0.90 ± 0.16	
23.0–24.9	990	0.89 ± 0.15	
≥25.0	1387	0.86 ± 0.17	
Education			
≤Elementary school	2126	0.84 ± 0.19	<0.001
Middle school	601	0.92 ± 0.12	
≥High school	1133	0.94 ± 0.10	
Income			
Quartile 1 (lowest)	919	0.85 ± 0.19	<0.001
Quartile 2	980	0.88 ± 0.17	
Quartile 3	976	0.89 ± 0.16	
Quartile 4 (highest)	985	0.91 ± 0.15	

Note: ^a^ *p* is determined by linear trend test.

**Table 3:** Descriptive characteristics of the participants by depression category.

Variable	All	Non-Depression	Depression
*n*	3860	3682	178
Age (years)	64.7 ± 9.1	64.7 ± 9.1	65.2 ± 8.4
BMI (kg/m^2^)	24.2 ± 3.3	24.2 ± 3.3	24.3 ± 3.1
EQ-5D	0.88 ± 0.17	0.89 ± 0.16	0.77 ± 0.23 *

Note: Data are mean ± standard deviation. * *p* < 0.0001 by *t*-test.

**Table 4:** Adjusted odds ratios (OR, 95% CIs) for disability for the EuroQol categories in postmenopausal women.

Dimension	Non-Depression (*n* = 3682)	Depression (*n* = 178)	*p*-Value
EuroQoL-mobility			
Model 1	1.00 (reference)	2.69 (1.94–3.72)	<0.001
Model 2	1.00 (reference)	2.49 (1.79–3.47)	<0.001
EuroQoL-self care			
Model 1	1.00 (reference)	2.66 (1.78–3.97)	<0.001
Model 2	1.00 (reference)	2.52 (1.68–3.78)	<0.001
EuroQoL-usual activities			
Model 1	1.00 (reference)	4.01 (2.91–5.54)	<0.001
Model 2	1.00 (reference)	3.86 (2.78–5.36)	<0.001
EuroQoL-pain/discomfort			
Model 1	1.00 (reference)	2.24 (1.64–3.05)	<0.001
Model 2	1.00 (reference)	2.14 (1.57–2.92)	<0.001
EuroQoL-anxiety/depression			
Model 1	1.00 (reference)	5.86 (4.30–7.99)	<0.001
Model 2	1.00 (reference)	5.52 (4.04–7.55)	<0.001

Note: Model 1 was adjusted for age; model 2 was adjusted for age, body mass index (BMI), educational level, and income.

## References

[1] Hidaka B.H. (2012). Depression as a disease of modernity: Explanations for increasing prevalence. J. Affect. Disord..

[2] Domènech-Abella J., Mundó J., Leonardi M., Chatterji S., Tobiasz-Adamczyk B., Koskinen S., Ayuso-Mateos J.L., Haro J.M. (2018). The association between socioeconomic status and depression among older adults in Finland, Poland and Spain: A comparative cross-sectional study of distinct measures and pathways. J. Affect. Disord..

[3] Djernes J.K. (2006). Prevalence and predictors of depression in populations of elderly: A review. Acta Psychiatr. Scand..

[4] Leach L.S., Christensen H., Mackinnon A.J., Windsor T.D., Butterworth P. (2008). Gender differences in depression and anxiety across the adult lifespan: The role of psychosocial mediators. Soc. Psychiatry Psychiatr. Epidemiol..

[5] Kessler R.C., McGonagle K.A., Zhao S., Nelson C.B., Hughes M., Eshleman S., Wittchen H.U., Kendler K.S. (1994). Lifetime and 12-month prevalence of DSM-III-R psychiatric disorders in the United States: Results from the National Comorbidity Survey. Arch. Gen. Psychiatry.

[6] Noble R.E. (2005). Depression in women. Metabolism.

[7] Steiner M., Dunn E., Born L. (2003). Hormones and mood: From menarche to menopause and beyond. J. Affect. Disord..

[8] Freeman E.W., Sammel M.D., Liu L., Gracia C.R., Nelson D.B., Hollander L. (2004). Hormones and menopausal status as predictors of depression in women in transition to menopause. Arch. Gen. Psychiatry.

[9] Westberg L., Eriksson E. (2008). Sex steroid-related candidate genes in psychiatric disorders. J. Psychiatry Neurosci..

[10] Ostlund H., Keller E., Hurd Y.L. (2003). Estrogen receptor gene expression in relation to neuropsychiatric disorders. Ann. N. Y. Acad. Sci..

[11] Prince M., Patel V., Saxena S., Maj M., Maselko J., Phillips M.R., Rahman A. (2007). No health without mental health. Lancet.

[12] Luppa M., Sikorski C., Luck T., Ehreke L., Konnopka A., Wiese B., Weyerer S., König H.H., Riedel-Heller S.G. (2012). Age- and gender-specific prevalence of depression in latest-life–systematic review and meta-analysis. J. Affect. Disord..

[13] Kessler R.C., Bromet E.J. (2013). The epidemiology of depression across cultures. Annu. Rev. Public Health.

[14] Ferrari A.J., Charlson F.J., Norman R.E., Patten S.B., Freedman G., Murray C.J., Vos T., Whiteford H.A. (2013). Burden of depressive disorders by country, sex, age, and year: Findings from the global burden of disease study 2010. PLoS Med..

[15] Ravindran A.V., Matheson K., Griffiths J., Merali Z., Anisman H. (2002). Stress, coping, uplifts, and quality of life in subtypes of depression: A conceptual frame and emerging data. J. Affect. Disord..

[16] Ritti-Dias R.M., Cucato G.G., de Matos L.D.N.J., Cendoroglo M.S., Nasri F., Wolosker N., Costa M.L.M., Gazelato de Mello Franco F. Depression and cancer were independently associated with quality of life in Brazilian older people. Australas. J. Ageing.

[17] Akosile C.O., Mgbeojedo U.G., Maruf F.A., Okoye E.C., Umeonwuka I.C., Ogunniyi A. (2018). Depression, functional disability and quality of life among Nigerian older adults: Prevalences and relationships. Arch. Gerontol. Geriatr..

[18] Akosile C.O., Anukam G.O., Johnson O.E., Fabunmi A.A., Okoye E.C., Iheukwumere N., Akinwola M.O. (2014). Fear of falling and quality of life of apparently-healthy elderly individuals from a Nigerian population. J. Cross Cult. Gerontol..

[19] Geerlings S.W., Beekman A.T.F., Deeg D.J.H., Twisk J.W.R., Van-Tilburg W. (2001). The longitudinal effect of depression on functional limitations and disability in older adults: An eight-wave prospective community-based study. Psychol. Med..

[20] Pennix B.W.J.H., Geerlings S.W., Deg D.J.H., van-Eijk J.T.M., van-Tilburg W., Beekman A.T.F. (1999). Minor and major depression and the risk of death in older persons. Arch. Gen. Psychiatry.

[21] Gureje O., Kola L., Afolabi E., Olley B.O. (2008). Determinants of quality of life of elderly Nigerians: Results from the Ibadan Study of Ageing. Afr. J. Med. Med. Sci..

[22] Jiang Y., Hesser J.E. (2006). Associations between health-related quality of life and demographics and health risks. Results from Rhode Island’s 2002 behavioral risk factor survey. Health Qual. Life Outcomes.

[23] Lorant V., Deliège D., Eaton W., Robert A., Philippot P., Ansseau M. (2003). Socioeconomic inequalities in depression: A meta-analysis. Am. J. Epidemiol..

[24] Dritsaki M., Petrou S., Williams M., Lamb S.E. (2017). An empirical evaluation of the SF-12, SF-6D, EQ-5D and Michigan Hand Outcome Questionnaire in patients with rheumatoid arthritis of the hand. Health Qual. Life Outcomes.

[25] Nam H.S., Kim K.Y., Kwon S.S., Koh K.W., Poul K. (2007). EQ-5D Korean Valuation Study Using Time Trade of Method.

[26] Lee S.I. (2011). Validity and Reliability Evaluation for EQ-5D in Korea.

[27] Bromet E., Andrade L.H., Hwang I., Sampson N.A., Alonso J., de Girolamo G., de Graaf R., Demyttenaere K., Hu C., Iwata N. (2011). Cross-national epidemiology of DSM-IV major depressive episode. BMC Med..

[28] Turgunova L., Laryushina Y., Turmukhambetova A., Koichubekov B., Sorokina M., Korshukov I. (2017). The incidence of depression among the population of central Kazakhstan and its relationship with sociodemographic characteristics. Behav. Neurol..

[29] Çakıcı M., Gökçe Ö., Babayiğit A., Çakıcı E., Eş A. (2017). Depression: Point-prevalence and risk factors in a North Cyprus household adult cross-sectional study. BMC Psychiatry.

[30] Lahelma E., Martikainen P., Laaksonen M., Aittomäki A. (2004). Pathways between socioeconomic determinants of health. J. Epidemiol. Community Health.

[31] Singh K., Kondal D., Shivashankar R., Ali M.K., Pradeepa R., Ajay V.S., Mohan V., Kadir M.M., Sullivan M.D., Tandon N. (2017). Health-related quality of life variations by sociodemographic factors and chronic conditions in three metropolitan cities of South Asia: The CARRS study. BMJ Open.

[32] Rezaei S., Hajizadeh M., Kazemi A., Khosravipour M., Khosravi F., Rezaeian S. (2017). Determinants of health-related quality of life in Iranian adults: Evidence from a cross-sectional study. Epidemiol. Health.

[33] Burström K., Johannesson M., Diderichsen F. (2001). Swedish population health-related quality of life results using the EQ-5D. Qual. Life Res..

[34] Terwindt G.M., Ferrari M.D., Tijhuis M., Groenen S.M., Picavet H.S., Launer L.J. (2000). The impact of migraine on quality of life in the general population: The GEM study. Neurology.

[35] Wahl A.K., Rustøen T., Rokne B., Lerdal A., Knudsen Ø., Miaskowski C., Moum T. (2009). The complexity of the relationship between chronic pain and quality of life: A study of the general Norwegian population. Qual. Life Res..

[36] Kind P., Dolan P., Gudex C., Williams A. (1998). Variations in population health status: Results from a United Kingdom national questionnaire survey. BMJ.

[37] Johnson J.A., Coons S.J., Ergo A., Szava-Kovats G. (1998). Valuation of EuroQOL (EQ-5D) health states in an adult US sample. Pharmacoeconomics.

[38] Hisashige A., Mikasa H., Katayama T. (1998). Description and valuation of health-related quality of life among the general public in Japan by the EuroQol. J. Med. Invest..

[39] Ghimire S., Baral B.K., Pokhrel B.R., Pokhrel A., Acharya A., Amatya D., Amatya P., Mishra S.R. (2018). Depression, malnutrition, and health-related quality of life among Nepali older patients. BMC Geriatr..

[40] Thau A.J., Rohn M.C.H., Biron M.E., Rahmatnejad K., Mayro E.L., Gentile P.M., Waisbourd M., Zhan T., Hark L.A. (2018). Depression and quality of life in a community-based glaucoma-screening project. Can. J. Ophthalmol..

[41] Akioyamen L.E., Genest J., Shan S.D., Inibhunu H., Chu A., Tu J.V. (2018). Anxiety, depression, and health-related quality of life in heterozygous familial hypercholesterolemia: A systematic review and meta-analysis. J. Psychosom. Res..

[42] Fu T., Cao H., Yin R., Zhang L., Zhang Q., Li L., Feng X., Gu Z. (2018). Depression and anxiety correlate with disease-related characteristics and quality of life in Chinese patients with gout: A case-control study. Psychol. Health Med..

[43] de Sousa R.D., Rodrigues A.M., Gregório M.J., Branco J.D.C., Gouveia M.J., Canhão H., Dias S.S. (2017). Anxiety and depression in the Portuguese older adults: Prevalence and associated factors. Front. Med..

[44] Sivertsen H., Bjørkløf G.H., Engedal K., Selbæk G., Helvik A.S. (2015). Depression and quality of life in older persons: A review. Dement. Geriatr. Cogn. Disord..

[45] Wariso B.A., Guerrieri G.M., Thompson K., Koziol D.E., Haq N., Martinez P.E., Rubinow D.R., Schmidt P.J. (2017). Depression during the menopause transition: Impact on quality of life, social adjustment, and disability. Arch. Women’s Ment. Health.

[46] Freeman E.W., Sammel M.D., Boorman D.W., Zhang R. (2014). Longitudinal pattern of depressive symptoms around natural menopause. JAMA Psychiatry.

[47] Bromberger J.T., Matthews K.A., Schott L.L., Brockwell S., Avis N.E., Kravitz H.M., Everson-Rose S.A., Gold E.B., Sowers M., Randolph J.F. (2007). Depressive symptoms during the menopausal transition: The study of women’s health across the nation (SWAN). J. Affect. Disord..

